# Introduction to the Special Issue

**DOI:** 10.31372/20200504.1125

**Published:** 2021

**Keywords:** Asian Pacific Islander Mental Health, Asian Americans, Pacific Islander, Mental Health issues, Epidemiology

This special issue includes seven articles. We have a brief review of mental health issues among Asian and Pacific Island (API) communities. Two papers examine non-biological factors that may be associated with mood disorders, using a nationally representative sample of Asian Americans (AA). These papers are particularly informative because they report disaggregated results across four AA subgroups. Another two articles examine different interventions and their efficacy to improve mood and quality of life among APIs. Finally, the case of a clinical encounter during COVID-19 was examined through the lens of Asian critical theory. Although these papers differ in their focuses and methodologies, collectively, they provide valuable information about mental health issues among the API community.

